# A dielectrophoretic method of discrimination between normal oral epithelium, and oral and oropharyngeal cancer in a clinical setting

**DOI:** 10.1039/c5an00796h

**Published:** 2015-06-09

**Authors:** K. A. Graham, H. J. Mulhall, F. H. Labeed, M. P. Lewis, K. F. Hoettges, N. Kalavrezos, J. McCaul, C. Liew, S. Porter, S. Fedele, M. P. Hughes

**Affiliations:** a Centre for Biomedical Engineering, Department of Mechanical Engineering Sciences, University of Surrey Guildford Surrey GU2 7XH UK m.hughes@surrey.ac.uk; b Faculty of Sports and Exercise Science, Loughborough University Loughborough Leicestershire LE11 3TU UK; c University College Hospital 235 Euston Rd London NW1 2BU UK; d Bradford Teaching Hospitals NHS Foundation Trust Duckworth Lane Bradford BD9 6RJ UK; e Head and Neck Centre University College Hospital Macmillan Cancer Centre Huntley Street London WC1E 6AG UK; f Eastman Dental Institute 256 Gray's Inn Road London WC1X 8LD UK

## Abstract

Despite the accessibility of the oral cavity to clinical examination, delays in diagnosis of oral and oropharyngeal carcinoma (OOPC) are observed in a large majority of patients, with negative impact on prognosis. Diagnostic aids might help detection and improve early diagnosis, but there remains little robust evidence supporting the use of any particular diagnostic technology at the moment. The aim of the present feasibility first-in-human study was to evaluate the preliminary diagnostic validity of a novel technology platform based on dielectrophoresis (DEP). DEP does not require labeling with antibodies or stains and it is an ideal tool for rapid analysis of cell properties. Cells from OOPC/dysplasia tissue and healthy oral mucosa were collected from 57 study participants *via* minimally-invasive brush biopsies and tested with a prototype DEP platform using median membrane midpoint frequency as main analysis parameter. Results indicate that the current DEP platform can discriminate between brush biopsy samples from cancerous and healthy oral tissue with a diagnostic sensitivity of 81.6% and a specificity of 81.0%. The present *ex vivo* results support the potential application of DEP testing for identification of OOPC. This result indicates that DEP has the potential to be developed into a low-cost, rapid platform as an assistive tool for the early identification of oral cancer in primary care; given the rapid, minimally-invasive and non-expensive nature of the test, dielectric characterization represents a promising platform for cost-effective early cancer detection.

## Abstract 2

DEP has the potential to be developed into a low-cost, rapid tool for the early identification of oral cancer in primary care.

## Graphical abstract

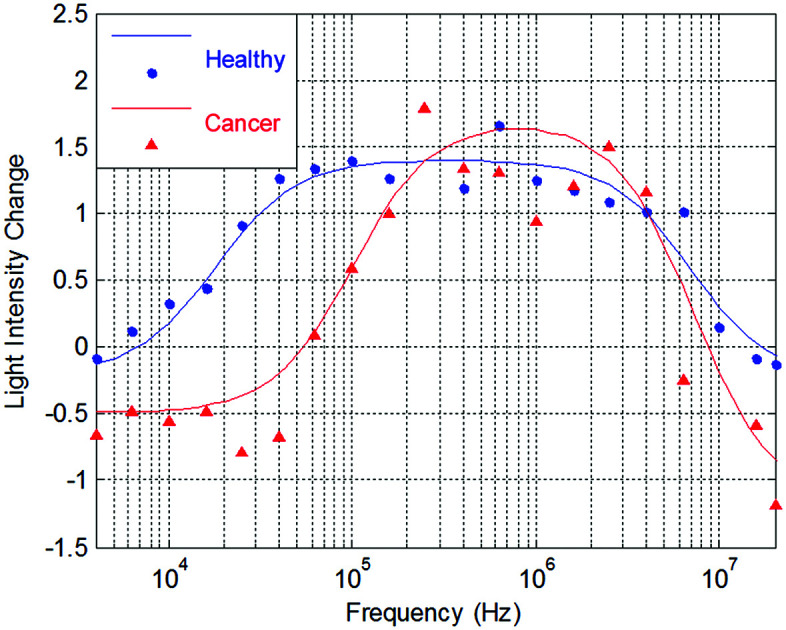

## Introduction

Head and neck cancer (HNC), primarily comprising squamous cell carcinomas of the oral cavity, oropharynx, and larynx, represents the 6^th^ most common cancer worldwide.^1^ The incidence of HNC is rising, mainly due to the increasing incidence of human papilloma virus (HPV)-related oropharyngeal carcinomas.^2^ Mortality rates remain high, with current data indicating that more than half of individuals with HNC die of their disease within five years of diagnosis.^3^ Most oral and oropharyngeal carcinomas (OOPCs) are preceded by long-standing clinical changes of the oral mucosa, mainly white (leukoplakia) and red (erythroplakia) patches.^4^ Prompt identification and histological examination of these mucosal abnormalities can translate into diagnosis of early-phase OOPC, *i.e.* at a stage of disease associated with favorable prognosis.^4^ However, early superficial OOPC is often difficult to clinically discern from common benign oral disease, and incisional biopsy is therefore always warranted to confirm the potentially malignant nature of oral lesions.^5^ Referral to secondary care for diagnostic incisional biopsy and subsequent histopathology is often delayed until the disease progresses to a more advanced, easier to detect but less curable stage, partially accounting for the high mortality of OOPCs.^4–7^

Currently a significant majority of patients present with advanced disease at diagnosis, despite the accessibility of the oral and oropharyngeal region to clinical examination, with relevant survival rates having remained at 20% or less over the last 20 years.^8,9^ A diagnostic system that could be used in a primary health care setting to identify early malignant disease could potentially translate into a prompt referral, earlier diagnosis and treatment of OOPC, and therefore theoretically improve prognosis.^7,10^ Published studies describing the implementation of non-invasive diagnostic aids (*e.g.* VELscope, Toluidine blue, Vizilite, OralCDx Brush Test)^6^ have thus far failed to demonstrate robust sensitivity and specificity in diagnosing OOPC, particularly early-stage disease and potentially-malignant lesions.^10–13^ Ideally, an early detection system for OOPC should consist of a non- or minimally-invasive, objective, fast, sensitive and specific, cost-effective test, conducted in a primary care setting to identify individuals with early disease, needing urgent referral to secondary cancer care.^14^

In recent years, alternating-current (AC) electrokinetic techniques, such as dielectrophoresis, have been employed to characterize and separate cells on the basis of their electrophysiological phenotypes.^15–17^ Dielectrophoresis (DEP) is observed when a polarizable particle, such as a cell, is subjected to a non-uniform electric field. The cell becomes motive under the influence of the dielectrophoretic force and is either attracted to or repelled by regions of high electric field gradient. The direction and magnitude of cell motion is governed, in part, by the electrophysiological properties of the cell and the frequency of the applied voltage; cells with different electrophysiological properties respond differently under the influence of the same range of frequencies, at the same applied voltage.^18^ Therefore, electrophysiological properties of cells can be inferred by analyzing cell motion over a range of frequencies and as DEP does not require labeling with antibodies or stains, it is an ideal tool for rapid analysis of cell properties, particularly in cases where biomarkers are not known or are not consistently expressed. DEP has been used in a variety of different fields within biomedicine, including numerous studies in cancer research. These studies have targeted a wide range of cancers, including colon, breast and ovarian cancers, contributing to the understanding of the mechanisms of carcinogenesis at a cellular level through investigation of cellular dielectric properties.^17,19–23^ Specifically within the field of oral cancer, DEP has been employed to characterize the electrophysiological properties of *in vitro* human OSCC cell lines *versus* immortalized keratinocyte cell lines.^24–26^

In this paper, we demonstrate that differences in electrophysiological profile exist between cells collected *via* oral brush biopsies (OBBs) from OOPC and healthy oral tissue. We demonstrate that a feature of DEP spectra produced by these OBB samples, the median midpoint frequency of the membrane dispersion, offers sensitivity and specificity (when comparing results from OOPC and healthy OBB samples) of 80% or greater. The aim was to demonstrate the feasibility of a simple and minimally-invasive means of detecting OOPC using a DEP-based system and a novel analysis parameter which eliminated the need to measure cell radius, and hence introduce a system of testing amenable to clinical translation. Our results indicate that with appropriate optimization, the DEP-based testing system presented in this paper offers a potential low cost-per-test detection method for oral cancer, with further potential cost reductions if samples were to be collected remotely and processed centrally.

## Materials and methods

### Study cohort and sample collection

Oral brush biopsies (OBBs) sampling OOPC and healthy tissue from the oral mucosa were collected from 57 recruited participants. OOPC OBB samples were collected from 45 consenting patients with clinical and histopathological features of OOPC, attending the Oral Medicine and Head and Neck Cancer clinics of the University College London Hospital NHS Foundation Trust (UCLHT) London, UK, and the Oral and Maxillofacial Surgery Department at Bradford Teaching Hospital, Bradford, UK. 21 healthy control OBB samples were collected from 12 participants (each participant providing no more than 2 samples), who were patients attending UCLHT Oral Medicine clinics for non-mucosal disease (*e.g.* dental or salivary gland disease and oro-facial pain). All experiments were performed in compliance with the relevant laws and institutional guidelines. The study received favourable ethical opinion by the National Research Ethics Service (NRES) Committee South East Coast – Kent (reference: 12/LO/1296. Project ID: 96530). All study participants provided written informed consent before recruitment into the study.

All OOPC OBB samples were collected in the period between initial diagnostic incisional biopsy and subsequent surgical resection, therefore histopathology was used as the gold standard for confirmation of the presence of OOPC tissue in the OBB samples. No incisional biopsy and associated histological confirmation was obtained for healthy control samples.

The minimally-invasive Rovers® Orcellex® Brush (Rovers Medical Devices B.V., The Netherlands) was selected for use in this study, due to availability of supply independent of a cytological analysis package. OBB sampling was performed by trained clinicians (SF, NK and JMcC), in accordance with the manufacturer's instructions for cell collection.^27^ The brush head, separated from the handle, was suspended in 5–6 ml sample storage medium, consisting of High Glucose (4.5 g L^−1^) DMEM (Biosera, East Sussex, UK) supplemented with 5 mL 100 units per ml penicillin and 100 μg ml^−1^ streptomycin (Sigma Aldrich, Poole, UK). Vials containing sampled specimens were labeled with an anonymized alpha-numeric code, and stored at 2–8 °C. All OBB samples were transported to the University of Surrey, Guildford for DEP testing.

A full DEP spectrum was successfully gained for 38 OOPC OBB samples, and 21 healthy OBB samples. 18 samples were rejected due to poor signal-to-noise ratio leading to low *R*-value in spectrum fit, caused either by insufficient cell yield for analysis and the presence of food, bacteria and other unidentified material within the sample.

### DEP sample preparation

Prior to DEP testing, a low-conductivity iso-osmotic DEP experimental medium was prepared, containing 17 mM dextrose and 248 mM sucrose in deionised water. The conductivity of this medium was then adjusted to 5 mS m^−1^ by addition of iso-osmotic phosphate-buffered saline solution, and verified using a Jenway 470 conductivity meter (VWR Jencons, Leicestershire, UK).

To prepare each OBB sample for DEP testing, the brush head was agitated in the surrounding storage medium using a vortex mixer on a low-speed setting, to dislodge any cells adhering to the brush bristles. The brush head was removed and the sample vial was placed in a Grant XB3 ultrasonic bath (Grant Instruments, Cambridgeshire, UK) for 1.5 minutes, to create a single cell solution. The resulting solution was filtered through a nylon mesh cell strainer, of pore size 100 μm (Fisher Scientific UK Ltd, Loughborough, UK), to remove cellular aggregates and keratin deposits, and the filter was subsequently flushed through with another 5 ml of the aforementioned storage medium.

The OBB sample cell solution was then centrifuged three times at 260*g* for 10 minutes; the first spin in sample storage medium and the subsequent two spins in fresh DEP experimental medium, to ensure removal of all traces of the highly-conductive sample storage medium. Following centrifugation, the OBB sample cell pellet was re-suspended in 200 μl of fresh DEP experimental medium.

Cell radii were estimated for each sample by capturing images of cells on a haemocytometer using an AVT Dolphin F145B digital camera (Allied Vision Technologies, Germany) mounted on a Nikon Eclipse microscope and connected to a PC, then analysed using Image J (National Institute of Mental Health, Maryland, US) image analysis software. The grid on a haemocytometer was used to scale the images.

### DEP-microwell experiments

DEP experiments were conducted using a prototype 3DEP (Deptech, Uckfield, UK) DEP-Well electrode chip and reader system, variations of which are explained in detail in prior publications.^26,28^ Each DEP experiment using this system quantified cell motion in response to application of a non-uniform AC electric field, by measuring change in light intensity distribution in the Well as a function of both time and distance (Well radius) for each frequency. The Well was divided into ten concentric rings for analysis; the four inner rings (in which no movement occurred during the analysis period) and the outer ring (which was emptied of cells by both positive and negative DEP) were not found to document cellular movement, and were therefore excluded from analysis to reduce the signal-to-noise ratio.

Five equally-spaced frequencies per decade (log scale) were used over a range of 4 kHz to 20 MHz to test each OBB sample and the DEP-Well electrode chip was energized by a 10 V peak–peak sinusoidal signal from a Digimess FG 100 function generator (Digimess, Reading, UK), whose output was monitored using ISO-Tech IDS710 digital oscilloscope. For each frequency tested, approximately 5 μl of cell solution was injected into a Well electrode on the DEP-Well chip, which was mounted above the light source of a Nikon Eclipse 50i upright microscope (Nikon, Surrey, UK) and viewed at 4× magnification. The aforementioned microscope-mounted digital camera, captured images of the cell solution in the Well electrode aperture area using Smart View for WDM software (version 0.1.3.3, supplied with the Allied Vision Technologies camera). Images were captured immediately prior to signal application (*i.e.* at time “zero” seconds) and every three seconds thereafter, for a period of 60 seconds. A MATLAB (the MathWorks Inc, Nantick, MA, USA) script was then used to assess the change in light intensity over the period that the electric current was applied and the change in light intensity was normalized to the image captured at time zero seconds. After each frequency was applied, the change in light intensity was plotted against frequency, to produce a DEP spectrum.

Prior to each frequency being tested, the DEP-well electrode was flushed clean using fresh DEP experimental medium and 5 μl of fresh OBB cell solution was injected. These steps ensured the results produced reflected the intrinsic frequency response of the cells, rather than the effects of prolonged cellular exposure to electric field.

### Statistical analysis

Statistical analysis of the study results was conducted using IBM SPSS Statistics version 19.0 (SPSS Inc. Chicago, IL). Receiver operating characteristic (ROC) curves were constructed to determine which analysis conditions yielded optimum results of sensitivity and specificity for the DEP-Well system. The mean and associated standard error of the mean, and the normality of both the OOPC and the healthy data were determined for each set of conditions. If both the OOPC and the healthy data set for each combination were normally distributed, a *T*-test was used to explore statistical significance. If one or both data sets were not normally distributed, the Mann-Whitney *U* test was used.

## Results and discussion

### Analysis of DEP spectra

**Fig. 1:**
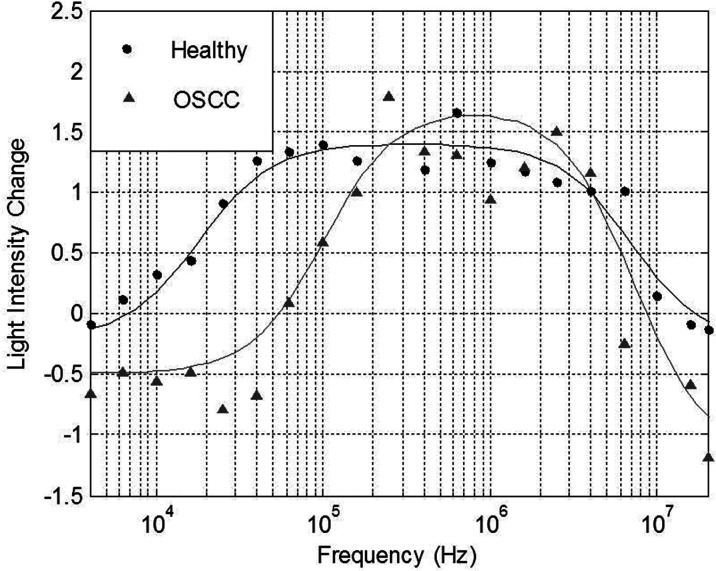
DEP spectra derived from healthy and OOPC samples, showing the effect of a change in median membrane midpoint frequency. Examples of electrophysiological spectra of exfoliated oral cells harvested from normal tissue and cancerous tissue are shown.

Change in light intensity *versus* frequency plots were constructed for each sample tested successfully using DEP. Upon examination of these DEP spectra, it became evident that differences between OOPC and healthy control OBB spectra occurred at the low frequency area of the plots, *i.e.* at frequencies ≤1 MHz.^29^ This indicated that differences between OOPC and healthy OBB samples were found at frequencies which cannot penetrate the cells’ plasma membranes, hence the movement of cells suspended in the DEP Well was indicative of the electrophysiological characteristics of the cells’ plasma membranes. Therefore, the characteristics of the membrane regions of the resulting DEP spectra were analyzed in greater detail. Electrophysiological spectra were obtained for exfoliated oral cells harvested from normal and abnormal tissue. Examples of the electrophysiological spectra of cells harvested from normal and abnormal tissue are shown in Fig. 1.

**Fig. 2:**
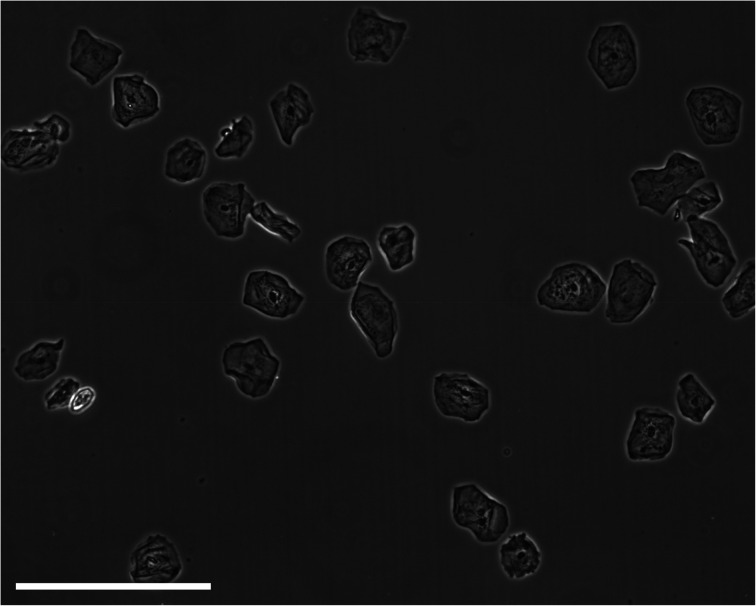
A micrograph of a typical exfoliated sample. The scale bar represents 100 μm.

Electrophysiological spectra for exfoliated oral cell specimens were grouped and spectra within each group were averaged. To average electrophysiological spectra, the light intensity change measured for each frequency point within the group was averaged. A “single-shell” model was used to approximate electrophysiological properties of cell suspensions from DEP spectra. However, the model assumes a spherical morphology with a lipid bilayer surrounding the cell interior.^21,30^ The morphology of keratinocytes (an example of an exfoliated sample can be seen in Fig. 2) is heterogeneous and dependent on cell maturation state; basal cells are spherical, prickle cells are cuboidal, granular cells are polyhedral and cornified cells are amorphic.^31^ Thus, exfoliated oral cell specimens do not comprise exclusively spherical cells. The majority of exfoliated oral cells are harvested from the external strata and are flat and amorphic. Depending on the site of harvest within the oral cavity, they may be parakeratinized (possessing a nucleus) or orthokeratinized (lacking a nucleus).^32^ Furthermore, keratinized cells in keratinized epithelium have a proteinacious layer deposited on the internal and external aspects of the cell membrane.^33^ However, the single-shell model was used to determine relative differences in cell electrophysiological properties. For each electrophysiological spectrum, the effective membrane capacitance value was determined using the single-shell model.^21^ The mean effective membrane capacitance of abnormal cell specimens (3.8 ± 1.0 mF m^−2^) was significantly lower than the mean effective membrane capacitance of normal cell specimens (6.5 ± 2.6 mF m^−2^) (*P* = 0.007). The presence of significant amounts of noise due to debris in the sample was responsible for large error margins; furthermore, both parameters fall below the capacitance value of ∼8 mF m^−2^ for a lipid bilayer, either because the structures do not possess lipid bilayers or because the heterogeneity of size and shapes of cells within the sample makes conventional modelling meaningless; cell radii ranged from approximately 9 μm for biconcave erythrocytes to approximately 55 μm for irregularly-shaped mature epithelial cells.

### Classification by median membrane midpoint frequency analysis

**Fig. 3:**
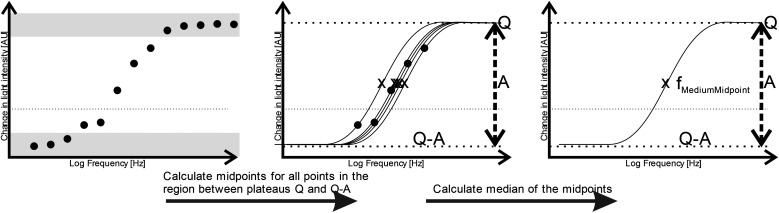
Schematic showing the process for determining the median membrane midpoint frequency for each DEP spectrum. Data points are classified according to whether they are within the top and bottom plateaus (dark grey bands) or the transition between the two (white band), according to whether they are within 10% of either end of the maximum displacement between lowest and highest value, For each point within the transition band, a curve is fitted perfectly through the point and its midpoint frequency *X* is calculated. The median value of the range of midpoint frequencies is the value selected.

Although the derived capacitance value indicated the presence of a statistically significant difference, analysis of individual samples and the use of capacitance as a cancer biomarker (*i.e.* using a capacitance value below a certain threshold as a classifier for cancer in a diagnostic test) did not provide results indicating that it could form the basis of an effective cancer test. We surmised that this may have been due both to the unusual morphology (and difficulty in generating meaningful radius data to use in the single-shell model) raised questions regarding the absolute efficacy of the model. In place of this, we examined the possibility identifying features in the shape of the DEP spectrum itself as a classifier. The capacitance value is associated with the frequency of the low-frequency dispersion (where the polarizability rises with increasing frequency), and so we examined whether the frequency at which this occurs could itself be used as a marker; a process shown schematically in Fig. 3.

Benguigui and Lin^34^ showed that the low frequency dispersion in Re[*K*(*ω*)]. can be approximated by the following expression:1
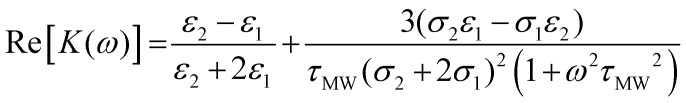
where *ε*_1_ and *ε*_2_ are the permittivities of the suspending medium and cells respectively, *σ*_1_ and *σ*_2_ are the conductivities of the suspending medium and cells respectively and *τ*_MW_ is the Maxwell-Wagner relaxation time, defined as:2
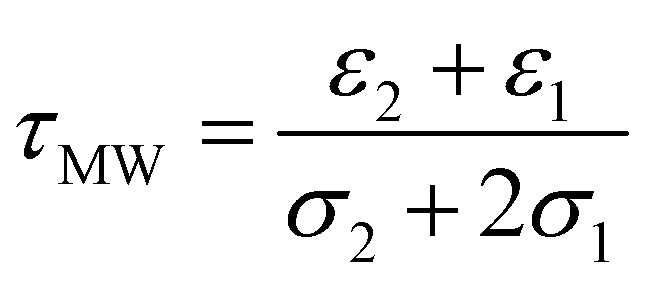


Eqn (1) can be rewritten and simplified to3
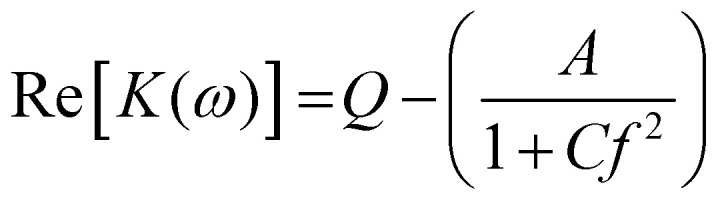
where *C* = (2π*τ*_MW_)^2^, *Q* is the polarizability at the high-frequency end of the dispersion, *A* is change in polarizability over the range of the dispersion. The midpoint of the transition (when Re[*K*(*ω*)] = *Q* − *A*/2) occurs when the condition in eqn (4) is met:4
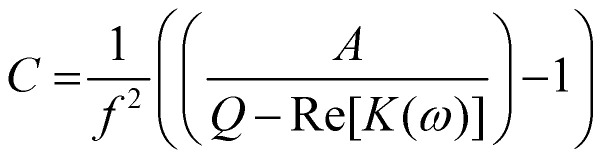


Substituting known values of *Q* and *A* from the DEP spectrum, plus a known value on the dielectric dispersion (a value of polarizability at a given measured frequency) allows *C* to be determined, and the characteristic midpoint frequency of the dispersion can be simply determined using5
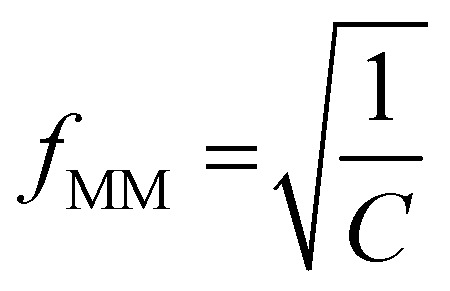


**Fig. 4:**
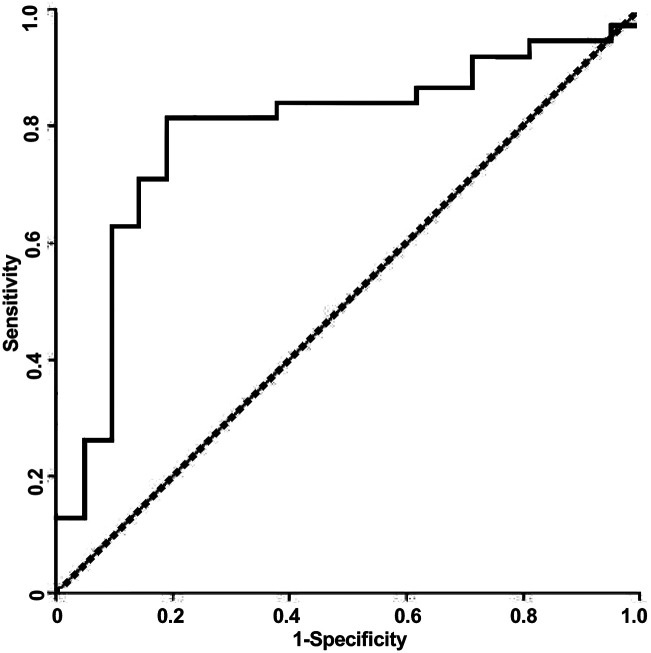
A Receiver-Operator Characteristic (ROC) curve for detection of OOPC using the DEP system described in the text.

Since the observed spectra contained a degree of noise and (unsurprisingly given the clinical nature of the samples) prone to occasional outliers, it was important to select a method of identifying a characteristic dispersion midpoint frequency that is not adversely affected by outliers. Average and best-fit strategies seek to find a best-fit to all data points, and as such gave unsatisfactory results. However, median fits are less susceptible to noise than means. Consequently, for each sample we generated curves through each of the data points observed to be in the dispersion (as opposed to at the initial or terminal plateaus), determined the midpoint frequency associated with each of these (typically, about five points are found in this region) curves, and selected the median of the five midpoint frequencies generated. The median of the membrane midpoint frequencies is then found for each OBB sample, to give the median membrane midpoint frequency, as shown in Fig. 3. In order to identify the data points in this transition region, points were assigned to the end transitions if they were within a certain percentage of the peak value at either end of the transition; we examined a range of band widths between 5–40% but found the optimum to be 10%, that is, samples were then considered to be part of the dispersion if they had values between 10–90% of the sweep of *A*. Analysis using this method produced a receiver-operator characteristic (ROC) curve (which shows the variation in sensitivity and specificity with the variation in frequency used for sample classification) as shown in Fig. 4. From this curve, the peak sensitivity and specificity were identified at 81.6% and 81.0%, respectively. When the descriptive statistics (mean ± standard error of the mean) were analyzed, the median membrane midpoint frequencies for OOPC OBB samples was found to be more than double that for healthy OBB samples (93.41 ± 10.91 kHz *vs.* 42.36 ± 6.07 kHz respectively). After confirming normality using the Kolmogorov-Smirnov test for normality, a student *T*-test demonstrated clear statistically significant differences (where *p* < 0.05) of *p* = 0.000. Although as indicated this may not correspond to the characteristic frequency of a particular cell types within a clearly heterogeneous sample, the effect is sufficiently consistent for the DEP analysis of the cellular ensemble has the potential to be used as a diagnostic marker; the values of sensitivity and specificity exceed those for the most common method of diagnosis (the conventional oral examination), and a combination of improved sample preparation with assay optimization (such as, for example, determining different thresholds for different areas of the mouth) may have the potential to boost this significantly.

## Conclusion

Establishing minimally-invasive methods for diagnosing OOPC is important for reducing the time to treatment and improving long-term outcomes of affected individuals; in particular, providing information to primary care clinicians to assist in assessing whether a patient presenting with an unidentified lesion should be referred to specialist secondary care for further investigation is critical in improving long-term survival. The present study sought to establish how effectively the DEP-Well system could distinguish between cells of oral brush biopsies (OBB) at the extreme ends of the tissue normality scale (*i.e.* OOPC and healthy), and therefore evaluate the possibility of using this system to recognize and hence diagnose OOPC.

This study presents a novel approach to the use of DEP for *ex vivo* cell analysis. The DEP-based platform was able to distinguish between OOPC and healthy *ex vivo* oral epithelial cells, sampled using oral brush biopsies, with a sensitivity of 81.6% and a specificity of 81% when classified according to the median values of the midpoint frequencies of the low-frequency dispersion. Furthermore, the low cost of the consumables in the assay (at time of writing, this was substantially below $10 per assay, and could be below $5 when purchased at scale) make the test amenable to wide-scale testing in primary care, even in relatively resource-poor areas. Furthermore, whilst the system used here required serial measurement using a DEP-well-based analysis platform, commercial parallel systems developed since this study was conducted would enable complete DEP spectra to be acquired in parallel, reducing assay time to one minute plus sample preparation. Cumulatively, these indicate that DEP has the potential to form the basis of a rapid, low cost OOPC tool for assisting clinicians when deciding whether to refer cases of concern to secondary care.

With automated, rapid sample handling, DEP has the potential for use at point of care; however, this work has also shown that samples are sufficiently robust to allow mail-in to centralized analysis laboratories, reducing the overhead costs. In order to meet this need, improved sample preparation is required to increase the number of biopsies producing a clinically useful result; studies are being undertaken to improve this.
